# COVID-19-Related and general racial discrimination and mental health in Asian Americans

**DOI:** 10.1186/s12889-025-24667-z

**Published:** 2025-10-09

**Authors:** Qiuxi Li, Harold Custodio, Karen Lim, James Jo, Salomeh Wagaw, Wendy Hetherington, Andrew M. Subica

**Affiliations:** 1 School of Medicine, University of California Riverside, Riverside, CA USA; 2 Asian Pacific Counseling and Treatment Centers, Special Service for Groups, Riverside, CA USA; 3 Riverside University Health System – Public Health, Riverside, CA USA

**Keywords:** Asian american, COVID-19, Racial discrimination, Mental health

## Abstract

**Background:**

Although reports indicate Asian Americans (AA) experienced racial discrimination during the COVID-19 pandemic, there exists a research gap that adequately details the nature and influence of racial discrimination on the mental health of AAs and key AA subgroups. This study aimed to capture the mental health and general racial and COVID-19-related discrimination experiences faced by AAs, and to examine whether discrimination associates with poor AA mental health outcomes.

**Methods:**

This study analyzed survey data collected between November 2022 and July 2023 from 447 AA adults who participated in a community-driven needs assessment conducted in partnership with Chinese, Korean, and Filipino community organizations in Riverside County, California. Mental health symptoms and discrimination experiences were assessed using validated instruments.

**Results:**

Data indicated that 16% and 15% of our AA participants screened positive for major depressive disorder and generalized anxiety disorder, respectively, with 38% reporting COVID-19-related discrimination. AA participants reported experiencing 1.6 instances of general racial discrimination across their lifetime, with Filipino AAs reporting significantly greater frequency of general discrimination vs. other AAs. Linear regressions revealed significant associations between depression and anxiety symptomology with age, fair/poor general health, and experiencing general racial—but not COVID-19-related—discrimination (*p <* .05).

**Conclusion:**

The study found that a notable proportion of AA respondents reported experiencing both COVID-19-related and general racial discrimination, and highlights the potential adverse impact of racial discrimination on AA mental health. These findings underscore the importance of examining and reducing anti-Asian discrimination experiences to improve the mental health and well-being of AA populations.

## Background

The COVID-19 pandemic led to a dramatic increase in racial discrimination directed towards Asian Americans (AA) of diverse heritages, who are commonly stereotyped as experiencing unparalleled economic, educational, and social success in the United States [1, 2]. Reports from across the United States documented a significant increase in discriminatory hate incidents targeting AAs, including physical assaults, verbal harassment, institutional bias, and discriminatory rhetoric on various media platforms [3–5]. For example, STOP AAPI Hate documented 11,409 reports of hate acts against AAs in the United States during the first two years of the pandemic [6]. While directly comparable pre-pandemic Hate incident data is not available, FBI crime statistics recorded 158 anti-Asian Hate crimes in 2019 [7], highlighting the sharp rise in reported acts of racial hostility during the pandemic. Such discriminatory acts were rooted in the misperception that individuals of Asian heritage were responsible for the spread of the SARS-CoV-2 virus, leading to heightened stigmatization and marginalization of AA communities [8].

Extensive literature has shown that experiencing racial discrimination can have detrimental effects on affected individuals’ mental health, increasing risk of depression, anxiety, and other psychological distress among discriminated individuals [9, 10]. Further, persistent exposure to discrimination, whether in interpersonal interactions, institutional settings, or within broader societal contexts, can create a toxic and hostile environment that erodes mental health and exacerbates feelings of helplessness and despair [11, 12]. The emotional toll of experiencing discrimination has also been linked to greater rates of social withdrawal, feelings of isolation, and diminished self-esteem [10, 11].

At the same time, some studies have suggested a nuanced relationship exists between discrimination and mental health. For instance, Cobb and his colleagues [13] conducted a study that indicated that the centrality of one’s ethnic/racial identity may moderate discrimination’s adverse effects on a person’s psychological well-being. However, while research examining discrimination and health has largely centered on racial/ethnic factors [14, 15], discrimination has also been shown to differently impact mental and physical health depending upon other moderating factors such as gender [16], age [17], weight [18], English proficiency [19], socioeconomic status [20], prior physical and mental health status [21], severity of discriminatory incidents [22], and whether experienced discrimination is chronic and persistent over time or an isolated event [23, 24]. Thus, racial discrimination’s impact on mental health may vary in strength depending on contextual and individual-level factors, including the nature of racial discrimination, the presence of moderator variables, and the population studied such as AAs.

For AAs, countering the “model minority” stereotype, AAs not only face a disproportionate burden of poor health outcomes and social disadvantage [25] but have endured systemic racial discrimination throughout their history in the United States [26, 27]. Additionally, the COVID-19 pandemic may have exacerbated disparities by exposing AAs to heightened levels of xenophobia, scapegoating, and violence [8]. This hostile environment has sparked a growing yet limited body of literature exploring the association between discrimination experiences and increased risk of mental health problems during the pandemic [28–30].

However, most studies on anti-Asian discrimination and mental health were conducted during the height of the pandemic in 2020 and 2021, with few studies examining whether these impacts persisted beyond the early stages of the pandemic. Moreover, limited research has explored how general racial discrimination and COVID-19-related discrimination may differ in their association with AA mental health. In the only known study to examine both types among AAs, Hahm et al. [31] used cross-sectional data collected in 2020 and found that COVID-related anti-Asian discrimination was associated with symptoms of posttraumatic stress, but not depression or anxiety. While both general and COVID-19-related discrimination reflect racial bias, COVID-19-related discrimination is a context-specific form of xenophobia tied to pandemic-associated scapegoating and disease stigma. In contrast, general racial discrimination reflects broader, chronic patterns of systemic and interpersonal racism. Distinguishing these allows us to examine whether pandemic-related discrimination had unique or overlapping mental health impacts beyond everyday racial stressors.

Understanding these distinctions also underscores the need to explore how such experiences may vary across Asian American subgroups—particularly given the inconsistent findings in prior studies and the tendency to treat AAs as a monolithic population in health data and research [26, 32, 33]. A major gap in particular exists regarding the heterogenous nature and influence of racial discrimination in AA subgroups vs. homogenized AA samples [34]. This gap poses a concern as AAs are the most diverse racial group in the United States—comprising over 20 nationalities with origins in East Asia, Southeast Asia/Philippines, South Asia, and Central Asia [35]. Despite this diversity, AAs remain persistently homogenized in extant research and health datasets; masking potentially important differences that may exist between AA subgroups [36]. These differences may reflect broader ethnoracial hierarchies, as certain subgroups report more frequent discrimination or have less access to protective social and structural resources. Consequently, scholars and advocates have highlighted the importance of, and need to, conduct disaggregated research to identify key health and mental health phenomena and patterns among AA subgroups [37–39].

To address these glaring literature gaps regarding the prevalence and nature of general racial and COVID-19-related discrimination in AAs, and its impact on AA mental health—including among major AA subgroups—this novel community-based participatory research (CBPR) project examined general racial and COVID-related anti-AA discrimination and their association with mental health in three of the four largest AA subgroups in the United States: Chinese, Korean, and Filipino AAs. Supported by the Centers for Disease Control and Prevention (CDC) and guided by a data disaggregation lens and our AA community partners, this study assessed AA adults across three domains: (1) sociodemographic characteristics and mental health outcomes (i.e., depression, anxiety, alcohol use disorder symptomology), (2) COVID-related and general racial discrimination, and (3) associations between these discrimination types and mental health outcomes.

## Methods

### Data collection

All protocols were approved by the University of California Riverside Institutional Review Board. Data were collected between November 2022 to July 2023 by trained community health workers (CHWs) as part of a county-wide needs assessment through collaborative CBPR partnerships with leading Chinese, Korean, and Filipino community organizations in Riverside County: the 10th largest county in the United States. Critically, following CBPR principles, this project emerged directly from requests by AA community organizations/allies to the Riverside public health department and CDC for support in examining and addressing urgent community concerns around rising COVID-19 discrimination, mental health difficulties, and need for increased vaccination outreach/engagement in Riverside’s historically underserved AA communities.

To design the survey for the needs assessment, the team conducted a workshop where the initial survey draft underwent an in-depth review and discussion with active participation from representatives from each partner AA community. This CBPR-based refinement process ensured the survey included topics of priority community interest, promoting cultural sensitivity and community engagement during data collection.

Adult participants were recruited by study CHWs through community-based convenience sampling at education outreach and community events—such as Chinese New Year celebrations— conducted in partnership with AA community organizations. Participation was entirely voluntary; refusal rates were not formally tracked, as individuals self-selected into the study after receiving information from CHWs. The CHWs, with linguistic and cultural backgrounds matching those of our AA participants, administered the survey in participant’s chosen language: English, Chinese, or Korean. Each survey participant received a $15 Amazon gift card upon survey completion.

### Measures

The survey assessed sociodemographics, mental health variables, alcohol consumption, and general racial and COVID-19-related discrimination experiences. Sociodemographic variables included age, gender, marital status, education, household income, English proficiency, and AA ethnicity.

For mental health outcomes, the survey administered two well-established screening tools: the Patient Health Questionnaire (PHQ-9) and the Generalized Anxiety Disorder scale (GAD-7). The PHQ-9 comprises nine questions designed to assess levels of depressive symptoms over the past two weeks, while the GAD-7 consists of seven questions assessing anxiety symptomology experienced over the past two weeks. Responses for both tools are scored on a scale from 0 to 3, ranging from “Not at all” (scored as 0) to “Nearly every day” (scored as 3). The total PHQ score ranges from 0 to 27, while the total GAD-7 score ranges from 0 to 21. A score equal to or greater than 10 was determined to be indicative of Major Depressive Disorder (MDD) for the PHQ-9 and Generalized Anxiety Disorder (GAD) for the GAD-7 [40, 41].

Alcohol consumption patterns were measured using the Alcohol Use Disorders Identification Test - Consumption (AUDIT-C). This tool comprises three items for identifying individuals engaging in hazardous drinking or experiencing alcohol use disorders (AUD). Each question within the AUDIT-C is rated on a scale from 0 to 4 points, resulting in a total score ranging from 0 to 12 points. A threshold score of ≥ 4 points for men and ≥ 3 points for women was identified as positive for probable AUD [42].

COVID-19-related discrimination was assessed using a set of descriptive items that follow a common structure widely used in national surveys on racial discrimination. Participants were first asked whether they had seen, heard, or experienced any COVID-19-related racism. Those who responded “Yes” were then asked to indicate their role in the incident (Target, Witness, Supporter, Other) and the type of discrimination involved (e.g., physical assault, verbal harassment, online harassment, being barred from public services, shunning, or other). While these items were not adapted from a formally validated psychometric scale, they align with established approaches for capturing specific, real-world experiences of discrimination in public health research [6]. Because the items were not designed to form a latent construct, internal consistency metrics (e.g., Cronbach’s alpha) for this measure were not applicable. For regression analyses, the binary variable of whether participants reported any experience with COVID-19-related discrimination (Yes = 1; No = 0) was used.

General racial discrimination experiences were assessed with the Experiences of Discrimination (EOD; [43]) scale, a widely-used instrument measuring exposure to a variety of routinely encountered discrimination experiences over their lifetime. The EOD assessed: (1) if participants Had experienced discrimination in 9 occasions: at school, getting hired or getting a job, at work, getting housing, getting medical care, getting service in a store or restaurant, on the street or in a public setting, and from the police or in the courts (yes = 1, no = 0), and (2) how often participants Had experienced discrimination in these situations with scores of 0 for ‘‘never,’’ 1 for ‘‘once,’’ 2.5 for ‘‘2–3 times,’’ and 5 to ‘‘4 or more times’’.

### Statistical analyses

Independent t-tests were conducted to assess mean differences between individuals who reported experiencing COVID-19-related discrimination and those who did not, as well as differences in experiences of general racial discrimination between AA subgroups. Chi-square tests were utilized to assess MDD and GAD prevalence between AA subgroups. Linear regression analyses were then employed to explore the associations between depressive and anxiety symptomology (dependent variables) and sociodemographic, general health, and general racial and COVID-19-related discrimination (independent variables). Binary variables were coded such that 0 represented the reference category (e.g., male, not married, non-heterosexual, English fluent, excellent/good general health) and 1 indicated the presence of the attribute (e.g., female, married, straight, not English fluent, fair/poor general health).

Assumptions of linear regression, including normality of residuals, homoscedasticity, and multicollinearity, were assessed using diagnostic plots and variance inflation factors (VIF). No major violations were observed and all VIF values were below 2.0, indicating no multicollinearity concerns. Missing data were minimal, with Less than 5% missing across key variables. Given the low level of missingness, listwise deletion was applied in all multivariate analyses. No systematic patterns of missingness were observed.

## Results

Table 1 presents the sample’s sociodemographic characteristics. The sample was composed of 447 AA adults: 209 of Korean, 136 of Chinese, and 102 of Filipino heritage. The majority of participants were women, married, and possessed a college degree, with notable variation across the three AA subgroups. For example, Filipino participants had the lowest percentage of married individuals and the highest percentage of college graduates while Korean participants had the largest proportion of households with incomes exceeding $100,000.

**Table 1:** Characteristics of survey participants

Characteristics	Asian Americans (*n* = 447)	Chinese Americans (*n* = 136)	Korean Americans (*n* = 209)	Filipino Americans (*n* = 102)
Women, %	60.8	61.0	55.8	69.3
Age, y, mean (SD)	54.2 (17.9)	48.4 (14.4)	59.8 (18.6)	50.2 (17.1)
Sex orientation, %				
Heterosexual	92.9	94.1	92.2	86.4
Non-heterosexual	3.9	2.2	2.4	8.6
Married or living as married, %	74.8	80.9	74.5	60.8
Education, %				
High school or less	18.4	23.4	19.1	10.2
Some college	18.6	18.8	19.1	17.3
Bachelor’s degree or higher	59.3	57.1	55.7	69.4
Household income, range, $, %				
< $40,000	20.8	19.4	23.4	17.8
$40,000-$99,999	33.3	37.7	28.0	38.0
$100,000+	19.7	13.2	25.5	16.5
Not English fluent, %	22.6	30.6	26.6	3.9
Dep. symptomology, mean (SD)	4.3 (5.1)	3.9 (5.1)	4.2 (5.0)	4.8 (5.6)
% (MDD)	16.4	14.4	15.9	20.2
Anxiety symptomology, mean (SD)	3.6 (5.0)	3.2 (5.2)	3.5 (4.9)	4.0 (5.2)
% (GAD)	15.2	12.9	14.3	17.9
Alcohol use disorder, mean (SD)	0.9 (1.8)	1.0 (1.7)	0.9 (1.9)	1.0 (1.6)
% (men ≥ 4, women ≥ 3)	10.7	11.0	11.0	9.8

*Dep*. Depression, *MDD* Major Depressive Disorder, *GAD* Generalized Anxiety Disorder

For mental health, participants reported moderate mean levels of depressive symptomology (*M* = 4.3), with 16% meeting the diagnostic threshold for MDD, and moderate mean levels of anxiety symptomology (*M* = 3.6), with 15% meeting the diagnostic threshold for GAD. Among AA subgroups, Filipino participants had the highest prevalence of screened MDD and GAD (20% and 18%, respectively), while Chinese participants had the lowest prevalence of MDD and GAD (14% and 12%, respectively). However, chi-square tests revealed no statistically significant differences (*p* <.05) across AA subgroups (MDD: χ² = 1.42, *p* =.49, Cramér’s V = 0.04; GAD: χ² = 0.79, *p* =.67, Cramér’s V = 0.04).

For alcohol, although AA participants showed relatively low levels of alcohol use (*M =* 1.0), 11% of participants met the diagnostic threshold indicating probable AUD. Korean and Chinese participants had the highest prevalence of probable AUD, suggesting potentially more frequent and/or heavier alcohol consumption patterns and an increased risk of alcohol-related harm within these subgroups.

Table 2 presents the prevalence of general racial and COVID-19-related discrimination experiences. More than one third of AA participants (38%) reported encountering COVID-19-related discrimination with 12% reporting personally experiencing COVID-19-related discrimination and 14% personally witnessing COVID-19-related discrimination toward others. The most frequently reported type of COVID-19-related discrimination was verbal harassment (28%), followed by physical assault (13%) and online harassment (9%). Independent t-test results indicated that participants who experienced any COVID-19-related discrimination had significantly greater depression (*M* = 5.6 vs. 3.4, *t* (259) = 4.0, *p* <.01, Cohen’s d = 0.46) and anxiety (*M =* 4.6 vs. 2.7, *t* (222) = 3.2, *p* <.01, Cohen’s d = 0.41) symptomology than participants who did not experience COVID-19-related discrimination.

**Table 2:** Discrimination experiences in the study sample

Characteristics	Asian Americans (%) (*n* = 447)	Chinese Americans (%) (*n* = 136)		Korean Americans (%) (*n* = 209)	Filipino Americans (%) (*n* = 102)	
COVID-19-Related Discrimination						
Have seen, heard, or experienced COVID-19-related racism incidents, %	38.4	33.8		40.5	40.4	
Role in the incident, %						
Target	11.6	7.4		13.6	13.7	
Witness	13.5	10.3		15.3	14.7	
Supporter	6.5	5.9		3.4	12.7	
Others	15.4	12.5		18.1	14.7	
Type of discrimination, %						
Physical assault	12.5	11.8		10.7	16.7	
Verbal harassment	27.5	19.9		30.5	32.4	
Online harassment	9.2	8.8		4.5	17.6	
Barred from public service	1.9	0.7		1.7	3.9	
Shunning	8.2	8.1		7.9	8.8	
Other	4.1	0.7		4.0	8.8	
General Racial Discrimination (EOD)						
Situations mentioned: EOD, 9 item						
0	70.5	79.4		71.8	55.9	
1–2	18.6	16.9		21.1	15.7	
3+	11	3.7		7.2	28.4	
Summary score: EOD, 9-item: mean (SD)						
Situation (possible range: 0–9)	0.8 (1.8)	0.4 (0.9)		0.6 (1.3)	1.9 (2.9)	
Frequency (possible range: 0–45)	1.6 (4.2)	0.7 (2.1)		1.1 (2.8)	3.9 (7.0)	
Cronbach’s alpha:						
EOD, situation	0.9	0.6		0.8	0.9	
EOD, frequency	0.9	0.6		0.7	0.9	

*EOD* Experiences of Discrimination Scale

For general racial discrimination, AA participants reported experiencing on average approximately 1 different type of discrimination in their lifetime (e.g., at school, getting service at stores). The most common locations for these experiences included the street (18%), store or restaurant (17%), school (13%), and the workplace (11%). On average, AA participants also reported experiencing 1.6 instances of general racial discrimination in their lifetime, with independent t-tests revealing significant differences between Filipino vs. Korean and Chinese participants. Specifically, Filipino participants reported experiencing more types of general racial discrimination over their lifetime than Korean (*M* = 1.9 vs. 0.6, *t* (309) = 5.4, *p <.*01, Cohen’s d = 0.66) and Chinese participants (*M* = 1.9 vs. 0.4, *t* (236) = 5.9, *p <.*01, Cohen’s d = 0.78). Additionally, they also reported a significantly higher frequency of general racial discrimination incidents over their lifetime than Korean (*M* = 3.9 vs. 1.1, *t* (309) = 4.9, *p <*.01, Cohen’s d = 0.60) and Chinese participants (*M* = 3.9 vs. 0.7, *t* (236) = 4.9, *p <*.01, Cohen’s d = 0.65).

Table 3 presents linear regression findings for the depression and anxiety models. In both models, age was significantly negatively associated with depression and anxiety symptomology, respectively, indicating that as age increased, depression and anxiety symptomology decreased (*p* <.05). Further, gender significantly associated with anxiety symptomology (*p <.*05), though not depression symptomology, indicating higher anxiety symptomology among female vs. male participants. Additionally, fair/poor general health significantly associated with depression and anxiety symptomology with having fair/poor general health associating with greater depression and anxiety symptomology (*p <.*05).

**Table 3:** Linear regressions of depression and anxiety symptomology by sociodemographic and discrimination factors

Dependent variables	Depression symptomology (PHQ-9)						Anxiety symptomology (GAD-7)				
	*b*		*SE*	*p-*value			*b*		*SE*	*p-*value	
Age	− 0.07^**^		0.02	< 0.00			− 0.11^**^		0.02	< 0.00	
Female	− 0.05		0.05	0.29			0.12^**^		0.04	0.01	
Straight	− 0.48		1.26	0.70			− 0.82		1.34	0.55	
Married	0.53		0.80	0.51			0.40		0.86	0.64	
Household income	− 0.05		0.19	0.78			0.13		0.20	0.53	
Education attainment	0.40		0.34	0.24			0.52		0.45	0.24	
Not English fluent	0.37		0.76	0.63			1.65		0.90	0.07	
Alcohol use	0.09		0.18	0.64			− 0.11		0.22	0.63	
Fair/poor general health	4.34^**^		0.71	< 0.00			3.72^**^		0.80	< 0.00	
COVID-related discrimination	0.01		0.01	0.98			− 0.01		0.01	0.51	
General racial discrimination	0.39^**^		0.08	< 0.00			0.25^**^		0.08	< 0.00	

*PHQ-9* Patient Health Questionnaire – 9, *GAD-7* Generalized Anxiety Disorder – 7, *b* Unstandardized Regression Coefficient, *SE* Standard Error

^*^p <.05

^**^p <.01

For the general racial and COVID-19-related discrimination, experiencing more types of general racial discrimination significantly associated with higher levels of both depression and anxiety symptomology (*p <.*05). However, no significant associations were found between COVID-19-related discrimination and depression and anxiety symptomology when controlling for sociodemographic variables, fair/poor health, and general racial discrimination.

## Discussion

Several key findings emerged from this novel community-requested and community-driven study of discrimination and mental health in AA communities. First, results revealed that a meaningful proportion of respondents reported experiencing: (1) COVID-19-related anti-Asian discrimination, and (2) general racial discrimination across their lifespan. Over one-third of AA participants reported experiencing racial discrimination related to the COVID-19 pandemic with verbal Harassment and physical assault the most commonly experienced forms. Additionally, over Half of our AA participants expressed concern about experiencing future incidents targeting either their personhood or their AA communities. On average, participants encountered 1.6 incidents of general racial discrimination in their lifetimes. These results indicate that a meaningful proportion of AA participants experienced COVID-19-related discrimination, and that racial prejudice remains a relevant concern within their lifetime. These findings are consistent with existing literature on discrimination in Asian American communities [4, 26, 27, 32, 44].

While limited research to date has examined discrimination among disaggregated AA subgroups, our results showed that Filipino Americans experienced heightened levels of general racial discrimination in their lifetime compared to Korean and Chinese Americans. This finding is partially consistent with Li’s work [45], which suggested that Filipino Americans were more likely to experience racial discrimination than other AA subgroups. Possible reasons for these differences may be: (1) Filipino Americans’ greater interracial contact (i.e., involvement with individuals from diverse racial backgrounds), and (2) lower intra-racial bonds (i.e., connections and solidarity within their own racial group) versus other AA subgroups [45].

Thus, our findings of AA subgroup differences in exposure to racial discrimination highlights the importance of further research on disaggregated AA subgroups. The elevated rates of depression and anxiety observed among Filipino participants may be partially explained by their greater exposure to discrimination—a noted finding for Filipino Americans in prior literature [46, 47], highlighting the importance of subgroup-specific analyses of mental health outcomes. These patterns may also reflect underlying demographic differences—such as a higher proportion of women in the Filipino subgroup—which were accounted for in our multivariable regression models.

With regard AA mental health, data indicated that participants reported potentially elevated prevalence of MDD (16%), and GAD (15%). While these rates are lower than those reported by scholars for AA adults during the early stages of the pandemic [31, 48], they are substantially higher than rates estimated for AA adults preceding the COVID-19 pandemic [49, 50]. Therefore, this potentially increased prevalence of MDD and GAD in our study sample suggests that while the mental health of AAs may have improved modestly from the pandemic’s peak, it remained elevated even at the later and post-stages of the COVID-19 pandemic, indicating a: 1) possible ongoing association between the COVID-19 pandemic and AA mental health, and (2) persistent need for attention to addressing the mental health needs of AA communities, particularly in the aftermath of the pandemic.

Interestingly, results indicated that participants reporting COVID-19-related discrimination exhibited elevated levels of depression and anxiety versus those not reporting COVID-19-related discrimination. As this finding is consistent with prior literature, it further supports the observed association between COVID-19-related discrimination and psychological well-being in AA adults [30, 48]. However, while this association was significant in bivariate t-tests, it was not significant in multivariable regression models. This discrepancy may be accounted for by other key covariates, including age, fair/poor health, and perhaps most critically, experiencing general racial discrimination across the lifetime. This pattern suggests that general racial discrimination may account for more variance in mental health outcomes when considered alongside pandemic-related discrimination.

This postulation is further bolstered by our regression findings of significant associations between general racial discrimination and depression and anxiety symptoms. Thus, our findings echo recent work that suggested that despite AAs being misperceived as a “model minority” in the United States, AAs mirror other racial minority groups as general racial discrimination appears to serve as a risk factor for adverse mental health outcomes in AAs [51]. Additionally, this finding aligns with the work of William and his colleagues [24] that indicated lifetime general racial discrimination events may have stronger association with mental health compared to acute experiences of discrimination (which may be reflected by COVID-19-related discrimination). Consequently, our findings may illuminate the important role that general racial discrimination may play in shaping the mental health of affected AAs.

Beyond advancing our knowledge of AA mental health and discrimination, these findings also offer practical implications for community organizations engaged in addressing anti-Asian discrimination and mental health disparities. As part of our CBPR framework, we shared disaggregated results with our local Chinese, Korean, Filipino, and county agency partners to support targeted outreach and programming. For instance, organizations serving Filipino American communities—who reported both higher discrimination exposure and elevated mental health symptoms—may consider prioritizing mental health screening and culturally tailored resources. Korean and Chinese partners have expressed interest in using study findings to inform stigma reduction strategies and expand language-accessible services. By providing subgroup-specific data, this study offered actionable insights that community stakeholders could apply in their ongoing community-based health equity and anti-discrimination efforts.

The limitations of this study should be acknowledged to provide a comprehensive understanding of the scope and implications of the research findings. First, the study relied on self-reported measures (e.g., PHQ-9, GAD-7) to assess mental health, which may be subject to recall bias, social desirability effects, and individual variation in symptom interpretation. However, both instruments are widely recognized and validated tools for assessing depressive and anxiety symptoms [40, 41].

Second, the Cronbach’s alpha for the EOD scale was lower than the commonly accepted threshold for the Chinese subgroup (α = 0.60–0.62), suggesting reduced internal consistency for this group. This may reflect cultural response patterns or differential interpretation of items. While the EOD is a widely used and validated instrument, subgroup comparisons involving Chinese participants should therefore be interpreted with caution. We also did not examine potential interaction effects between our study variables and AA subgroups, and our sample size was not powered to detect subgroup-by-discrimination interaction effects, which would require substantially larger samples to assess with adequate statistical precision. Future research with larger samples may therefore wish to build on this work and examine potential interactions across diverse AA subgroups including several not assessed in this study such as South Asian and Southeast Asian subgroups.

Third, because participants were recruited from Riverside County, the findings may not be generalizable to broader AA populations in the U.S. Nevertheless, the study provides valuable insights into the mental health impacts of discrimination within an underserved and diverse Asian American population.

Finally, the demographic composition of our sample—primarily middle-aged and female—reflects the nature of our community-based recruitment strategy, which was implemented through culturally tailored outreach at public events and organizational programs. As such, while the sample provides valuable insight into community-engaged AAs, the findings may be less generalizable to younger or male-dominated AA populations who may be underrepresented in such outreach settings and in convenience samples.

## Conclusion

In conclusion, this study provides significant insights into the scope and nature of racial discrimination experienced by AA communities—both generally and in response to the COVID-19 pandemic—and the association between these experiences and AA mental health, with a particular focus on Chinese, Korean, and Filipino communities. Specifically, study findings indicate a concerning prevalence of MDD and GAD among AAs, which persisted despite potential modest improvements from the peak of the pandemic, and their frequent experiences with racial discrimination experienced by AAs, both generally and in relation to the COVID-19 pandemic. Overall, our findings support the need for greater attention to studying mental health disparities and combating racial discrimination within AA communities, with implications for both research and practice.

## Data Availability

The datasets generated and analyzed in this study are available from the corresponding author on reasonable request.

## References

[1] Chou RS, Feagin JR. Myth of the model minority: Asian Americans facing racism. New York: Routledge; 2015.

[2] Yi V, Museus SD. Model minority myth. The Wiley Blackwell encyclopedia of race, ethnicity, and nationalism. Hoboken: Wiley-Blackwell; 2015;1-2.

[3] Gutierrez AM, Schneider SC, Islam R, Robinson JO, Hsu RL, Canfield I, et al. Experiences of stigma in the United States during the COVID-19 pandemic. Stigma and Health. 2024;9(2):103-111. 10.1037/sah0000354.

[4] Ha SK, Nguyen AT, Sales C, Chang RS, Ta H, Srinivasan M et al. Increased self-reported discrimination and concern for physical assault due to the COVID-19 pandemic in Chinese, Vietnamese, Korean, Japanese, and Filipino Americans. Journal of Asian Health. 2021;9;e202105. 10.59448/jah.v1i1.5.

[5] Schild L, Ling C, Blackburn J, Stringhini G, Zhang Y, Zannettou S. Go eat a bat, chang! An early look on the emergence of Sinophobic behavior on web communities in the face of covid-19. arXiv preprint. 2020;arXiv:2004.04046.

[6] Community Reports to Stop AAPI Hate 2020–2022 key findings.2023. https://stopaapihate.org/wp-content/uploads/2023/10/23-SAH-TaxonomyReport-KeyFindings-F.pdf.

[7] Federal Bureau of Investigation. 2019 Hate Crime Statistics. U.S. Department of Justice. 2020. https://ucr.fbi.gov/hate-crime/2019

[8] Devakumar D, Shannon G, Bhopal SS, Abubakar I. Racism and discrimination in COVID-19 responses. Lancet. 2020;395:1194.10.1016/S0140-6736(20)30792-3 32246915 10.1016/S0140-6736(20)30792-3 PMC7146645

[9] Mays VM, Cochran SD, Barnes NW. Race, race-based discrimination, and health outcomes among African Americans. Annu Rev Psychol. 2007;58:201–25. 16953796 10.1146/annurev.psych.57.102904.190212 PMC4181672

[10] Pascoe EA, Smart Richman L. Perceived discrimination and health: a meta-analytic review. Psychol Bull. 2009;135:531. 19586161 10.1037/a0016059 PMC2747726

[11] Brondolo E, Hausmann LR, Jhalani J, Pencille M, Atencio-Bacayon J, Kumar A, et al. Dimensions of perceived racism and self-reported health: examination of racial/ethnic differences and potential mediators. Ann Behav Med. 2011;42:14–28. 21374099 10.1007/s12160-011-9265-1 PMC4973890

[12] Schmitt MT, Branscombe NR, Postmes T, Garcia A. The consequences of perceived discrimination for psychological well-being: a meta-analytic review. Psychol Bull. 2014;140:921. 24547896 10.1037/a0035754

[13] Cobb CL, Meca A, Branscombe NR, Schwartz SJ, Xie D, Zea MC, et al. Perceived discrimination and well-being among unauthorized Hispanic immigrants: the moderating role of ethnic/racial group identity centrality. Cult Divers Ethn Minor Psychol. 2019;25:280. 10.1037/cdp0000227 30284850

[14] Pachter LM, Caldwell CH, Jackson JS, Bernstein BA. Discrimination and mental health in a representative sample of African-American and Afro-Caribbean youth. J Racial Ethn Health Disparities. 2018;5:831–7. 28916954 10.1007/s40615-017-0428-z PMC5854514

[15] Wallace S, Nazroo J, Bécares L. Cumulative effect of racial discrimination on the mental health of ethnic minorities in the United Kingdom. Am J Public Health. 2016;106:1294–300. 27077347 10.2105/AJPH.2016.303121 PMC4984732

[16] Lee JH, Gamarel KE, Bryant KJ, Zaller ND, Operario D. Discrimination, mental health, and substance use disorders among sexual minority populations. LGBT Health. 2016;3:258–65. 27383512 10.1089/lgbt.2015.0135 PMC4976222

[17] Yuan ASV. Perceived age discrimination and mental health. Soc Forces. 2007;86:291–311.

[18] Emmer C, Bosnjak M, Mata J. The association between weight stigma and mental health: a meta-analysis. Obes Rev. 2020;21:e12935. 31507062 10.1111/obr.12935

[19] Spencer MS, Chen J, Gee GC, Fabian CG, Takeuchi DT. Discrimination and mental health–related service use in a national study of Asian Americans. Am J Public Health. 2010;100:2410–7. 20299649 10.2105/AJPH.2009.176321 PMC2978178

[20] Stepanikova I, Oates GR. Perceived discrimination and privilege in health care: the role of socioeconomic status and race. Am J Prev Med. 2017;52:S86–94. 27989297 10.1016/j.amepre.2016.09.024 PMC5172593

[21] Bogart LM, Wagner GJ, Galvan FH, Landrine H, Klein DJ, Sticklor LA. Perceived discrimination and mental health symptoms among black men with HIV. Cult Divers Ethn Minor Psychol. 2011;17:295. 10.1037/a0024056 PMC3148695 21787061

[22] Monroe SM. Modern approaches to conceptualizing and measuring human life stress. Annu Rev Clin Psychol. 2008;4:33–52. 17716038 10.1146/annurev.clinpsy.4.022007.141207

[23] Cohen S, Murphy ML, Prather AA. Ten surprising facts about stressful life events and disease risk. Annu Rev Psychol. 2019;70:577–97. 29949726 10.1146/annurev-psych-010418-102857 PMC6996482

[24] Williams DR, Neighbors HW, Jackson JS. Racial/ethnic discrimination and health: findings from community studies. Am J Public Health. 2003;93:200–8. 12554570 10.2105/ajph.93.2.200 PMC1447717

[25] Office of Minority Health. Profile: Asian Americans. U.S. Department of Health and Human services; 2019. https://minorityhealth.hhs.gov/asian-american-health.

[26] Gee GC, Ro A, Shariff-Marco S, Chae D. Racial discrimination and health among Asian americans: evidence, assessment, and directions for future research. Epidemiol Rev. 2009;31(1):130–51. 19805401 10.1093/epirev/mxp009 PMC4933297

[27] McMurtry CL, Findling MG, Casey LS, Blendon RJ, Benson JM, Sayde JM, et al. Discrimination in the United States: experiences of Asian Americans. Health Serv Res. 2019;54(S2):1419–30. 31657465 10.1111/1475-6773.13225 PMC6864377

[28] Le PD, Misra S, Hagen D, Wang SM, Li T, Brenneke SG, et al. Coronavirus disease (COVID-19) related discrimination and mental health in five US Southern cities. Stigma Health. 2023;8:133.

[29] Raj A, Chatterji S, Johns NE, Yore J, Dey AK, Williams DR. The associations of everyday and major discrimination exposure with violence and poor mental health outcomes during the COVID-19 pandemic. Soc Sci Med. 2023;318:115620. 36587480 10.1016/j.socscimed.2022.115620 PMC9750505

[30] Wu C, Qian Y, Wilkes R. Anti-Asian discrimination and the Asian-white mental health gap during COVID-19. Ethnic and Racial Studies. 2021;44(5):819-835.

[31] Hahm HC, Ha Y, Scott JC, Wongchai V, Chen JA, Liu CH. Perceived COVID-19-related anti-Asian discrimination predicts post traumatic stress disorder symptoms among Asian and Asian American young adults. Psychiatry Res. 2021;303:114084. 34242971 10.1016/j.psychres.2021.114084 PMC8661065

[32] Gee GC, Spencer MS, Chen J, Takeuchi D. A nationwide study of discrimination and chronic health conditions among Asian Americans. Am J Public Health. 2007;97:1275–82. 17538055 10.2105/AJPH.2006.091827 PMC1913081

[33] Jung MY, Juon H-S, Slopen N, He X, Thomas SB, Lee S. Racial discrimination and health-related quality of life: an examination among Asian American immigrants. J Racial Ethn Health Disparities. 2022;9:1262–75. 34086197 10.1007/s40615-021-01067-8 PMC8176876

[34] Mereish EH, Liu MM, Helms JE. Effects of discrimination on Chinese, Pilipino, and Vietnamese Americans’ mental and physical health. Asian Am J Psychol. 2012;3:91.

[35] Budiman A, Ruiz N. Key facts about Asian americans, a diverse and growing population. Pew Res Cent. 2021. https://www.pewresearch.org/short-reads/2021/04/29/key-facts-about-asian-americans/

[36] Subica AM, An K, Okamoto SK. Asian American and native hawaiian/pacific islander substance use and disparities: review of current evidence and recommendations for the field. Curr Addict Rep. 2024. 10.1007/s40429-024-00544-4.

[37] Ponce NA, Shimkhada R, Adkins-Jackson PB. Making communities more visible: equity-centered data to achieve health equity. Milbank Q. 2023;101:302–32. 37096622 10.1111/1468-0009.12605 PMC10126976

[38] Shimkhada R, Scheitler A, Ponce NA. Capturing racial/ethnic diversity in population-based surveys: data disaggregation of health data for Asian American, Native Hawaiian, and Pacific Islanders (AANHPIs). Popul Res Policy Rev. 2021;40(1):81–102.

[39] Teranishi RT, Nguyen BMD, Alcantar CM. The Asian American and Pacific Islander data disaggregation movement: the convergence of community activism and policy reform. Asian Am Policy Rev. 2014;25:26.

[40] Kroenke K, Spitzer RL, Williams JB. The PHQ-9: validity of a brief depression severity measure. J Gen Intern Med. 2001;16:606–13. 11556941 10.1046/j.1525-1497.2001.016009606.x PMC1495268

[41] Spitzer RL, Kroenke K, Williams JB, Löwe B. A brief measure for assessing generalized anxiety disorder: the GAD-7. Arch Intern Med. 2006;166:1092–7. 16717171 10.1001/archinte.166.10.1092

[42] Bradley KA, DeBenedetti AF, Volk RJ, Williams EC, Frank D, Kivlahan DR. Audit-C as a brief screen for alcohol misuse in primary care. Alcohol Clin Exp Res. 2007;31:1208–17. 17451397 10.1111/j.1530-0277.2007.00403.x

[43] Krieger N, Smith K, Naishadham D, Hartman C, Barbeau EM. Experiences of discrimination: validity and reliability of a self-report measure for population health research on racism and health. Soc Sci Med. 2005;61(7):1576–96. 16005789 10.1016/j.socscimed.2005.03.006

[44] Zhou S, Banawa R, Oh H. Stop Asian hate: the mental health impact of racial discrimination among Asian Pacific Islander young and emerging adults during COVID-19. Health Serv Res. 2021;56(S2):8–9. 10.1016/j.jad.2022.12.132 36623563

[45] Li M. Discrimination and psychiatric disorder among Asian American immigrants: A National analysis by subgroups. J Immigr Minor Health. 2014;16:1157–66. 24077835 10.1007/s10903-013-9920-7

[46] Nadal KL, Vigilia Escobar KM, Prado GT, David E, Haynes K. Racial microaggressions and the Filipino American experience: recommendations for counseling and development. J Multicult Couns Dev. 2012;40:156–73.

[47] Nadal KLY, Corpus G, Hufana A. The forgotten Asian americans: Filipino americans’ experiences with racial microaggressions and trauma. Asian Am J Psychol. 2022;13:51.

[48] Liu CH, Zhang E, Wong GTF, Hyun S. Factors associated with depression, anxiety, and PTSD symptomatology during the COVID-19 pandemic: clinical implications for US young adult mental health. Psychiatry Res. 2020;290:113172. 32512357 10.1016/j.psychres.2020.113172 PMC7263263

[49] Sangalang CC, Gee GC. Depression and anxiety among Asian Americans: the effects of social support and strain. Soc Work. 2012;57:49–60. 22768628 10.1093/sw/swr005

[50] Sung SC, Low CCH, Fung DSS, Chan YH. Screening for major and minor depression in a multiethnic sample of A Sian primary care patients: A comparison of the nine-item patient health questionnaire (PHQ‐9) and the 16‐item quick inventory of depressive Symptomatology–Self‐Report (QIDS‐SR16). Asia‐Pacific Psychiatry. 2013;5:249–58. 24123813 10.1111/appy.12101

[51] Lee YH, Liu Z, Fatori D, Bauermeister JR, Luh RA, Clark CR, et al. Association of everyday discrimination with depressive symptoms and suicidal ideation during the COVID-19 pandemic in the all of us research program. JAMA Psychiatr. 2022;79:898–906. 10.1001/jamapsychiatry.2022.1973 PMC9330278 35895053

